# Linkages between COVID-19, solar UV radiation, and the Montreal Protocol

**DOI:** 10.1007/s43630-023-00373-w

**Published:** 2023-03-30

**Authors:** G. H. Bernhard, S. Madronich, R. M. Lucas, S. N. Byrne, T. Schikowski, R. E. Neale

**Affiliations:** 1 Biospherical Instruments Inc., San Diego, CA USA; 2 Atmospheric Chemistry Observations and Modeling Laboratory, National Center for Atmospheric Research, Boulder, USA; 3 National Centre for Epidemiology and Population Health, Australian National University, Canberra, Australia; 4 Faculty of Medicine and Health, The University of Sydney, School of Medical Sciences, Sydney, Australia; 5 Leibniz Research Institute for Environmental Medicine, Düsseldorf, Germany; 6 Population Health Program, QIMR Berghofer Medical Research Institute, Brisbane, Australia; 7 School of Public Health, University of Queensland, Brisbane, Australia

## Abstract

There are several connections between coronavirus disease 2019 (COVID-19), solar UV radiation, and the Montreal Protocol. Exposure to ambient solar UV radiation inactivates SARS-CoV-2, the virus responsible for COVID-19. An action spectrum describing the wavelength dependence of the inactivation of SARS-CoV-2 by UV and visible radiation has recently been published. In contrast to action spectra that have been assumed in the past for estimating the effect of UV radiation on SARS-CoV-2, the new action spectrum has a large sensitivity in the UV-A (315–400 nm) range. If this “UV-A tail” is correct, solar UV radiation could be much more efficient in inactivating the virus responsible for COVID-19 than previously thought. Furthermore, the sensitivity of inactivation rates to the total column ozone would be reduced because ozone absorbs only a small amount of UV-A radiation. Using solar simulators, the times for inactivating SARS-CoV-2 have been determined by several groups; however, many measurements are affected by poorly defined experimental setups. The most reliable data suggest that 90% of viral particles embedded in saliva are inactivated within ~ 7 min by solar radiation for a solar zenith angle (SZA) of 16.5° and within ~ 13 min for a SZA of 63.4°. Slightly longer inactivation times were found for aerosolised virus particles. These times can become considerably longer during cloudy conditions or if virus particles are shielded from solar radiation. Many publications have provided evidence of an inverse relationship between ambient solar UV radiation and the incidence or severity of COVID-19, but the reasons for these negative correlations have not been unambiguously identified and could also be explained by confounders, such as ambient temperature, humidity, visible radiation, daylength, temporal changes in risk and disease management, and the proximity of people to other people. Meta-analyses of observational studies indicate inverse associations between serum 25-hydroxy vitamin D (25(OH)D) concentration and the risk of SARS-CoV-2 positivity or severity of COVID-19, although the quality of these studies is largely low. Mendelian randomisation studies have not found statistically significant evidence of a causal effect of 25(OH)D concentration on COVID-19 susceptibility or severity, but a potential link between vitamin D status and disease severity cannot be excluded as some randomised trials suggest that vitamin D supplementation is beneficial for people admitted to a hospital. Several studies indicate significant positive associations between air pollution and COVID-19 incidence and fatality rates. Conversely, well-established cohort studies indicate no association between long-term exposure to air pollution and infection with SARS-CoV-2. By limiting increases in UV radiation, the Montreal Protocol has also suppressed the inactivation rates of pathogens exposed to UV radiation. However, there is insufficient evidence to conclude that the expected larger inactivation rates without the Montreal Protocol would have had tangible consequences on the progress of the COVID-19 pandemic.

**Graphical abstract:**

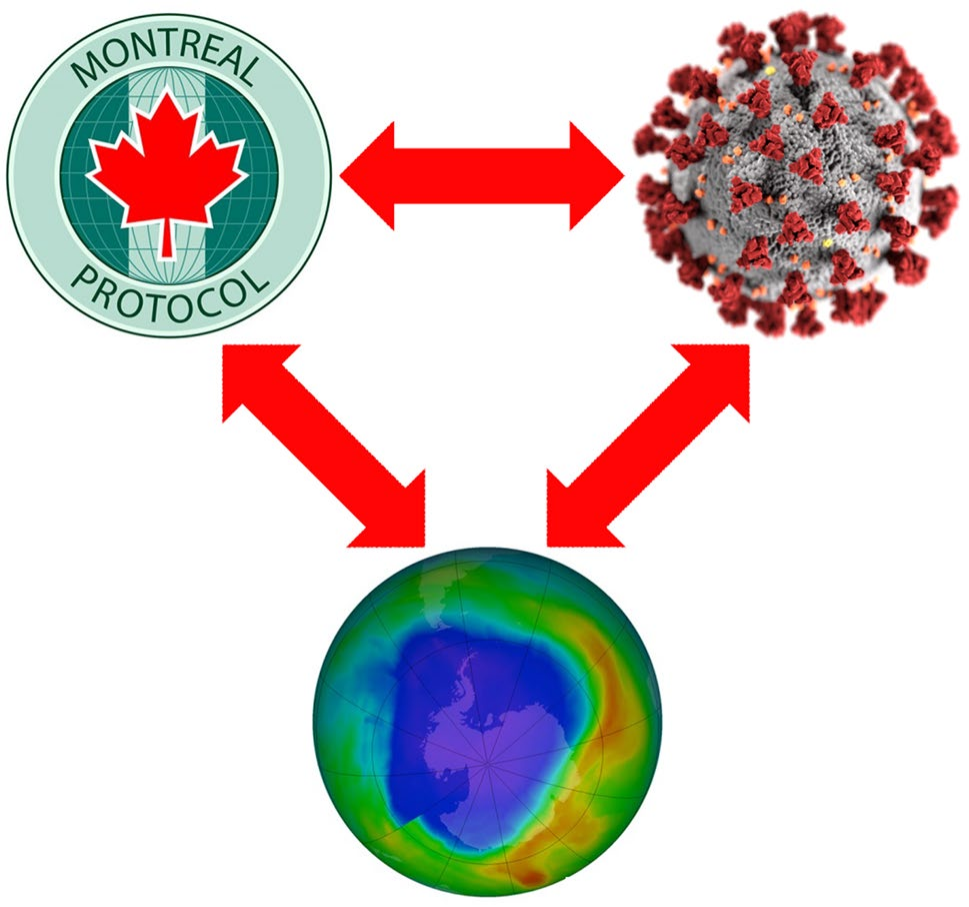

## Introduction

This Perspective is part of the topical collection: Environmental effects of stratospheric ozone depletion, UV radiation, and interactions with climate change: UNEP Environmental Effects Assessment Panel, 2022 Quadrennial Assessment (10.1007/s43630-023-00374-9). Coronavirus disease 2019 (COVID-19) is an infectious disease caused by severe acute respiratory syndrome coronavirus 2 (SARS-CoV-2). It was first identified in December 2019 in China and has resulted in a global pandemic. The 2020 Assessment Update [1] by the Environmental Effects Assessment Panel (EEAP) of the Montreal Protocol under the United Nations Environment Programme (UNEP) included a section on linkages between COVID-19, solar ultraviolet (UV) radiation, and the Montreal Protocol. At the time the publication was prepared, COVID-19 had been in existence for less than 1 year and research on the subject was incomplete with many papers still in review. The scientific literature is now more mature and we present an update on the current knowledge of the aspects of COVID-19 related to solar UV radiation and the Montreal Protocol.

SARS-CoV-2 is mainly transmitted from person to person through large respiratory droplets and aerosols (small droplets with diameters ≤ 5 μm [2]) generated by breathing, talking, sneezing, singing, and coughing in close proximity to another person [3–7]. The relative role of large droplets vs aerosols is still unclear [8], but aerosols can penetrate more deeply into the lungs than droplets [2]. Indirect transmission through fomites (defined as inanimate objects carrying pathogens) that have been contaminated by respiratory secretions is considered possible [9], but research suggests that this path of transmission is unlikely [4, 10–12].

A 2021 review article concluded, based on 5 studies, that less than 10% of globally reported SARS-CoV-2 infections occurred outdoors [13]. The odds of indoor transmission was 18.7 (95% confidence interval (CI) 6.0, 57.9) times higher compared to outdoor transmission. Furthermore, in high- and middle-income countries, about 92% of time is spent indoors or in a vehicle [14]. Hence, it would be expected that more than 90% of transmissions occur indoors, even if the likelihood of in- and outdoors transmission were equal. These studies support the currently prevailing view that most transmissions occur indoors where there is essentially no exposure to solar UV-B (280–315 nm) radiation and greatly reduced exposure to ambient UV-A (315–400 nm) radiation.

Many publications (see Sect. 5) have provided evidence for an inverse relationship between ambient solar UV radiation and incidence or severity of COVID-19. However, the reasons for this negative correlation^1^ are not clear. The following hypotheses may explain this association:

1. Exposure to ambient solar UV radiation inactivates SARS-CoV-2 particles, and higher intensity of UV radiation (e.g. at latitudes closer to the Equator) is more effective in inactivating the virus.
2. UV radiation is merely a proxy for other environmental factors such as air temperature that are responsible for the observed inverse correlation. For example, when UV radiation is low in winter, temperature is often also low, prompting humans to stay indoors in close proximity to others, thereby increasing the chance for SARS-CoV-2 transmission. Lower temperatures or other environmental factors such as air pollution may also compromise the immune system and may have a detrimental effect on disease outcome [15].
3. Higher intensity of UV-B radiation leads to more production of vitamin D (Sect. 6) or other substances produced in the skin upon exposure to UV radiation, such as nitric oxide, which may have benefits for disease prevention and severity.

These three hypotheses will be discussed in the following subsections.

Many experimental studies (often using unrealistically large virus concentrations) have shown that SARS-CoV-2 particles can remain viable on porous and non-porous surfaces for several days if they are shielded from UV radiation [16–20]. Survival times on porous surfaces are generally much shorter than on impermeable surfaces because of the different evaporation mechanisms for the two surface types [21]. Infectious SARS-CoV-2 viruses have been recovered from plastic, glass, and stainless steel surfaces after 3 days [16], 7 days [17] and 28 days [18]. SARS-CoV-2 virus particles have been shown to remain viable on banknotes for between four [17] and up to 28 days when the ambient temperature was maintained at 20 °C [18]. On the outer layer of a surgical mask, infectious viruses can survive up to 6 days after contamination. Increasing the ambient temperature greatly reduces the survivability of virus particles on all surfaces to as little as 24 h at 40 °C [18]. According to a recent study [22], the Alpha, Beta, Delta, and Omicron variants of SARS-CoV-2 exhibit more than two-fold longer survival on plastic and skin than the original Wuhan strain. As we will show below, these long lifetimes of SARS-CoV-2 particles decrease greatly upon exposure to UV radiation.

## The action spectrum for the inactivation of SARS-CoV-2

Action spectra describe the wavelength dependence of biological effects caused by UV radiation. A biological effect is quantified by first multiplying the action spectrum for this effect with the spectrum of the incident radiation and then integrating this product over wavelength. The result is the biologically effective UV irradiance, UV_BE_.

The action spectrum for the inactivation of SARS-CoV-2 has recently been measured by Biasin et al. [23] using light-emitting diode (LED) sources with ~ 10 nm bandwidth at wavelengths of 254, 278, 308, 366, and 405 nm. The experiment for establishing the action spectrum has several weaknesses: the uncertainties of the measurements at these wavelengths were not evaluated; the LED’s bandwidth of 10 nm is large for measuring a function that varies over four decades; and interpolating measurements at only 5 wavelengths over the 150 nm wide wavelength range of interest is subject to large interpolation errors that have not been discussed. While these limitations are significant, we emphasise that these are the only measurements of the action spectrum for the inactivation of SARS-CoV-2 that are available to date (August 2022).

**Fig. 1:**
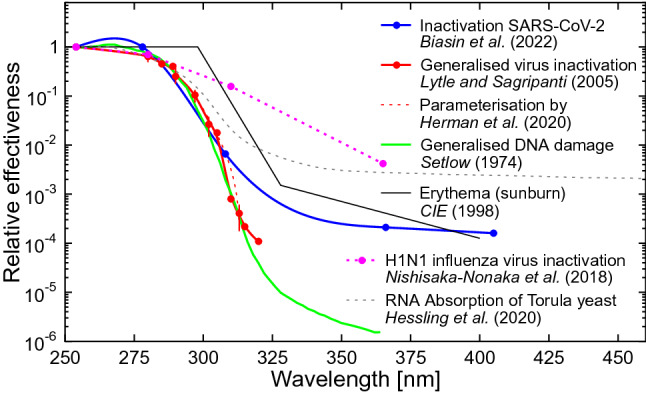
Comparison of action spectra and other spectra relevant for discussing the wavelength dependence of inactivation of SARS-CoV-2 by UV radiation. All spectra are arbitrarily normalised at 254 nm. Biasin et al. [23] measured the relative effectiveness of UV radiation in inactivating SARS-CoV-2 at 278, 308, 366, and 405 nm (blue symbols). Data were interpolated with a spline approximation that also included a data point at 254 nm measured by Biasin et al. [32]. Note that this interpolation is uncertain between 280 and 305 nm due to the lack of intermediate measurements in this wavelength range. Lytle and Sagripanti [24] give the generalised action spectrum for virus inactivation (red symbols). The spectrum is based on data from up to 24 viruses (data at some wavelengths are based on fewer viruses). Data were interpolated with a spline approximation with little uncertainty (red line). The broken red line is a fit to the same data by Herman et al. [33] using an analytical function. Note that this function deviates significantly from the data points between 307 and 312 nm. Setlow [34] provides the generalised action spectrum for DNA damage as parameterised in the Tropospheric Ultraviolet and Visible (TUV) radiative transfer model (https://www.acom.ucar.edu/Models/TUV/Interactive_TUV/) (green line). CIE [28] shows the action spectrum for erythema (black line). The action spectrum from Nishisaka-Nonaka et al. [27] is for the inactivation of the H1N1 influenza virus by inhibiting replication and transcription of viral RNA in host cells (pink symbols). The spectrum from Heßling et al. [26] is not an action spectrum but the absorption spectrum of RNA of Torula yeast, normalised at 254 nm. (Absorption and action spectra would be identical if every absorbed photon were to lead to irreversible damage; however, this is not the case if repair mechanisms are at play [35].)

Figure 1 compares the action spectrum by Biasin et al. [23] with action spectra that have been used previously to estimate the effect of UV radiation on SARS-CoV-2. Of note, the new spectrum has a large sensitivity in the UV-A (315–400 nm) range, while the spectrum for generalised virus inactivation [24]—which has been used in several studies (discussed below) to estimate the inactivation times of SARS-CoV-2—has no sensitivity beyond 320 nm. If the “UV-A tail” measured by Biasin et al. [23] is correct, solar UV radiation could be much more efficient in inactivating the virus responsible for COVID-19, and inactivation would be less influenced by parameters that affect the UV-A and UV-B contributions to solar radiation differently, such as total column ozone^2^ (TCO), time of the day, season, or latitude. While this large UV-A tail is missing in the generalised action spectrum for virus inactivation [24], there is evidence that this sensitivity in the UV-A range is real. First, the recently measured absorption spectrum of RNA of Torula yeast also has a large contribution from the UV-A range [26]. Second, the H1N1 influenza virus also seems to be highly sensitive to radiation in the UV-A range [27], and even the action spectrum for erythema [28] has a remarkable resemblance to that measured by Biasin et al. [23]. Third, the dependence of the inactivation times of SARS-CoV-2 on UV spectra simulated for various solar zenith angles (SZA) discussed below can only be explained if there is a significant contribution from UV-A wavelengths. This argument is also supported by theoretical calculations [29]. Fourth, it has recently been shown that exposing human coronavirus 229E (CoV-229E)—a virus associated with a range of respiratory symptoms including pneumonia and bronchiolitis—to UV-A radiation leads to a significant reduction in coronavirus spike protein and decreased virus-induced death of infected human tracheal epithelial cells [30]. Fifth, it has also been shown that many viruses can be damaged by peroxides and other reactive oxygen species, which are created by UV-A radiation [31]. However, whether a similar oxidative toxicity also affects SARS-CoV-2 has not yet been determined. Taken together, these considerations suggest that UV-A-mediated mechanisms in addition to RNA damage [26], which is predominantly caused by UV-B wavelengths, lead to the inactivation of SARS-CoV-2 upon exposure to UV radiation.

## Inactivation times of SARS-CoV-2 virus particles with solar UV radiation

Several studies have determined the time needed to inactivate 90% of virus particles upon irradiation with UV radiation, based on: (i) direct measurements using solar simulators [36–41]; (ii) the action spectrum for the inactivation of SARS-CoV-2 particles measured by Biasin et al. [23], followed by theoretical calculations; or (iii) theoretical calculations using the standardised action spectrum for virus inactivation [33, 42–44]. These studies are discussed in more detail below and results are summarised in Table 1.

**Table 1:** Time to inactivate 90% (10% survival) of virus particles at 40° N upon irradiation with UV radiation

Study	Time *t*_10_ = time to inactivate 90% of infectious virus (minutes)				Ratio
Measured inactivation times					
	21-Jun (Summer) SZA = 16.6° UV-B = 1.83 W m^−2^ UV-A = 58.5 W m^−2^ UVI = 10.2	7-Mar SZA = 45.1 UV-B = 0.92 W m^−2^ UV-A = 40.5 W m^−2^ UVI = 4.8	21-Feb SZA = 50.8 UV-B = 0.70 W m^−2^ UV-A = 34.9 W m^−2^ UVI = 3.6	21-Dec (Winter) SZA = 63.4 UV-B = 0.28 W m^−2^ UV-A = 21.5 W m^−2^ UVI = 1.6	Winter/Summer
Saliva on steel [37]	6.8		8.0	12.8	1.9
Growth medium (gMEM) [37]	14.3		17.6	54.4	3.8
Aerosol in saliva [36]	7.5	19			
Aerosol in culture medium [36]	12.6	13.6			
Calculated inactivation times					
Calculated by us, based on the action spectrum by [23] and *D*_10_ inactivation dose of 8.1 J m^−2^ at 254 nm	4.4	7.4	9.1	16.5	3.8
Calculated by us, based on the action spectrum by Lytle and Sagripanti [24] and *D*_10_ inactivation dose of 3.2 J m^−2^ at 254 nm	6.1	18.1	27.2	97.2	15.9
Calculated by Herman et al. [33]. Based on the action spectrum by Lytle and Sagripanti [24] as parameterised by Herman et al. [33] and *D*_10_ inactivation dose of 3.2 J m^−2^ at 254 nm	4.8	13.4	19.8	69.3	14.6
Calculated by Sagripanti and Lytle [43]. Based on the action spectrum by Lytle and Sagripanti [24] and D_10_ inactivation dose of 6.9 J m^−2^ at 254 nm	22	63*		> 300	> 14

*Refers to spring equinox on 21 March instead of 7 March

All studies assume that the number of viable virus particles, $N$, decreases exponentially when exposed to germicidal radiation (either from artificial light sources or the Sun) for the time $t$ [45]:

1 $$N = N_{0} e^{ - \alpha t}$$

where $N_{0}$ is the number of viable particles at the start of the exposure and $\alpha$ is a decay constant. With $t_{10}$ and $t_{1}$ defined as the times that reduce $N$ to 10% and 1% of $N_{0}$, respectively, Eq. (1) implies that $t_{1}$ is twice as long as $t_{10}$. However, the time difference between $t_{10}$ and $t_{1}$ can be considerably longer under certain conditions [46, 47] because viruses in a real-world setting are embedded in a matrix of body fluids (e.g. saliva and mucus) or foreign objects, which partially shield viruses from exposure. Clustered populations of viruses can also protect each other from exposure to radiation [48]. Hence, there are large uncertainties in extrapolating $t_{10}$ to $t_{1}$ and beyond (e.g. 0.1% and 0.0001% survival for disinfection and sterilisation levels, respectively [44]). This is particularly the case for virus particles that are embedded in porous materials, such as face masks and clothing, or otherwise shielded from UV radiation.

### Measured inactivation times

Ratnesar-Shumate et al. [37] used a solar simulator to determine $t_{10}$ for SARS-CoV-2 virus particles that were first suspended in either simulated saliva or a culture medium (gMEM^3^) and then dried on stainless steel surfaces. The solar simulator produced spectral irradiance resembling noon-time solar spectra at 40° N latitude (e.g. Philadelphia, Ankara, Beijing) for three days representative of summer, spring, and winter: 21 June (SZA = 16.5°), 21 February (SZA = 50.6°) and 21 December (SZA = 63.4°). The three spectra were compared with spectra modelled with the Tropospheric Ultraviolet and Visible (TUV) radiative transfer model (https://www.acom.ucar.edu/Models/TUV/Interactive_TUV/), and the agreement was generally excellent. However, the simulated spectrum for 21 June (high sun conditions in summer of the Northern Hemisphere) underestimated the spectrum calculated by TUV at wavelengths less than 308 nm. This difference is significant because the product of the solar spectrum and any action spectra for virus inactivation typically peaks near 305 nm, but the consequence of this discrepancy cannot be quantitatively assessed from the data provided by Ratnesar-Shumate et al. [37]. Inactivation times $t_{10}$ for virus particles embedded in saliva were 6.8, 8.0 and 12.8 min for the three spectra, respectively. For viruses enclosed in the culture medium, $t_{10}$ was more than twice as long: 14.3, 17.6, and 54.4 min for the spectra simulated for 21 June, 21 February, and 21 December, respectively.

Using the same solar simulator, Schuit et al. [36] determined $t_{10}$ for aerosolised virus particles. For viruses suspended in saliva, $t_{10}$ was 7.5 and 19 min for exposure to simulated noon solar spectra for 21 June (SZA = 16.5°) and 7 March (SZA = 45.0°), respectively.

In addition to the two studies discussed above, Sloan et al. [41] determined inactivation times for SARS-CoV-2 using another solar simulator (SunLite Solar Simulator Model 11,002 from Abet Technologies). The simulator was set to “1 Sun”, defined as “full sunlight intensity on a bright clear day on Earth and measuring approximately 1000 W m^−2^”. According to data provided by the manufacturer, the simulator produces UV-A and UV-B irradiances of 41.46 W m^−2^ and 1.28 W m^−2^, respectively, at these settings. These irradiances are reportedly similar to those measured at the equinox at 40° N latitude during noon. However, the authors do not show a spectrum of their solar simulator and we, therefore, could not determine whether the device does indeed simulate the solar spectrum accurately, in particular in the critical wavelength range of 300–320 nm. Viral solution was suspended in either culture medium or simulated mucus and then deposited on stainless steel coupons, and desiccated. For virus suspended in culture medium, the inactivation time $t_{10}$ was 23 min under controlled temperature (22.5 °C) and relative humidity (RH = 34%). When the virus was suspended in simulated mucus, the inactivation time was significantly longer ($t_{10}$ = 91 min). These inactivation times are longer than those measured by Ratnesar-Shumate et al. [37]; however, a direct comparison is not possible because of the uncertainty of the solar spectrum used by Sloan et al. [41]. While the study confirms that inactivation times depend on the medium enclosing the virus, the results contradict those by Ratnesar-Shumate et al. [37], which indicate shorter inactivation times for virus embedded in saliva versus a growth medium.

In another study, Raiteux et al. [39] irradiated stainless steel coupons loaded with a suspension containing SARS-CoV-2 virus with a solar simulator consisting of a Xenon lamp and a filter. The simulator was set to an illuminance^4^ of either 10,000 lx, representing “a cloud-covered sky in autumn in France”, or 56,000 lx, representing “a slightly cloudy sky in summer in France”. No further description of the simulator’s output is given and it is unknown whether the spectrum resembles that of sunlight in the UV-B and UV-A regions. Results of the study should, therefore, be considered only in a qualitative sense. The experiment revealed that no virus was detectable after a 20 min exposure to an illuminance of 10,000 lx at either 20 or 35 °C and a relative humidity of 50%. For an illuminance of 56,000 lx, infectious virus was no longer detectable after 5 min of exposure. Ninety percent of viral load was lost every 9.2 min at 10,000 lx and every 2.1 min at 56,000 lx. The inactivation time was inversely proportional to the applied illuminance within the measurement uncertainty. This suggests that the UV-B and UV-A contributions of the lamp spectra scale linearly with illuminance. The results are qualitatively consistent with those by Ratnesar-Shumate et al. [37] and confirm that simulated sunlight rapidly inactivates SARS-CoV-2 at temperatures ranging from 20 to 35 °C.

Using a model 91293 solar simulator from Oriel Instruments, which was equipped with a filter to block visible and infrared radiation, Wondrak et al. [38] confirmed that “UV radiation at environmentally relevant doses” will inactivate SARS-CoV-2 coronaviruses. However, no inactivation times were calculated and the study, therefore, cannot be used for a quantitative assessment.

### Calculated inactivation times

All studies discussed in the previous section used a solar simulator to determine inactivation times. In contrast, Carvalho et al. [44], Herman et al. [33], Nicastro et al. [40], and Sagripanti and Lytle [43] used an indirect method to estimate $t_{10}$ based on the inactivation dose at 254 nm (a wavelength in the UV-C waveband produced by a mercury lamp) and an action spectrum for virus inactivation. The inactivation time $t_{10}$ in minutes is then calculated from these quantities:

2 $$t_{10} [\min ] = \frac{{D_{10} (\lambda_{r} )}}{{60s \times E_{e} }} = \frac{{D_{10} (\lambda_{r} )}}{{60s \times \int {E(\lambda )A(\lambda )d\lambda } }}$$

where $D_{10} (\lambda_{r} )$ is the UV dose at 254 nm that results in 10% survival, $A(\lambda )$ is the action spectrum, and $E(\lambda )$ is the spectral irradiance of sunlight modelled for various SZAs and TCO. The integral is evaluated over a wavelength range where both $E(\lambda )$ and $A(\lambda )$ are different from zero. Of the three studies, the most reliable is the one by Nicastro et al. [40] because it uses the measured action spectrum by Biasin et al. [23] for the inactivation of SARS-CoV-2 particles and $D_{10} (\lambda_{r} )$ measured also for SARS-CoV-2. In contrast, Herman et al. [33] and Sagripanti and Lytle [43] use the action spectrum by Lytle and Sagripanti [24]. (See Fig. 1 for comparison of action spectra).

Using the action spectrum and dose $D_{10} (254)$ reported by Nicastro et al. [40], we calculated inactivation times of 3.5 and 12.2 min for noon-time spectra on the summer solstice (21 June), and on the winter solstice (21 December) for northern latitudes of 40°. For similar conditions, Sagripanti and Lytle [43] calculate considerably longer times of 22 min and > 300 min because calculations are based on the action spectrum by Lytle and Sagripanti [24], which does not have a UV-A contribution (Fig. 1). Using the same action spectrum, Herman et al. [33] calculated inactivation times $t_{10}$ for viruses adhered to fomites oriented horizontally under clear skies. For SZAs of less than 20°, 40°, and 60°, inactivation times were less than 8, 20, and 60 min, respectively. These times are generally smaller than those determined by Sagripanti and Lytle [43], mostly due to the difference in the inactivation dose at 254 nm assumed by the two studies. We note that the calculations by Herman et al. [33] are also affected by their poor parameterisation of the action spectrum (Fig. 1). Likewise, the interpolation of the measurements by Nicastro et al. [40] is uncertain because the action spectrum was measured at only five wavelengths with a relatively large bandwidth of ~ 10 nm (Sect. (2)). We further note that the ratio of inactivation times for winter and summer (last column of Table 1) calculated by Nicastro et al. [40] agree much better with the times measured by Ratnesar-Shumate et al. [37] than the times calculated by Herman et al. [33] and Sagripanti and Lytle [43]. This observation is strong evidence that the action spectrum by Biasin et al. [23], with its large UV-A tail, is closer to the actual action spectrum.

Carvalho et al. [44] calculated inactivation times for locations across the globe based on UV radiation data from the Tropospheric Emission Monitoring Internet Service (TEMIS), which uses assimilated UV radiation fields from several space-borne instruments. These UV radiation data refer to the daily radiant exposure and were weighted with the DNA action spectrum [34] shown in Fig. 1. Hence, the effect from UV-A wavelengths was likely underestimated. In contrast to the studies discussed above, Carvalho et al. [44] took into account that virus particles embedded in aerosol are equally sensitive to radiation from all directions. They assumed inactivation doses $D_{10} (254)$ of 1.8 J m^−2^ (least conservative, based on [36]) and 7.0 J m^−2^ (most conservative, based on [37]), and calculated inactivation times $t_{10}$ of ~ 5 min for overhead Sun (e.g. São Paulo (24° S), Brazil) for the least conservative scenario. During summer in Iceland, $t_{10}$ was calculated to range between 30 and 100 min. These inactivation times are similar in magnitude to those listed in Table 1. Carvalho et al. [44] performed similar calculations for sterilisation level inactivation, where the fraction of “surviving” viruses is less than one millionth of the initial number of viable virus particles. Associated sterilisation times are naturally much longer than $t_{10}$; however, these times are of little practical use considering that neither the number of viable particles at the start of the exposure nor the number of virus particles that result in an infection is well known.

In summary, the uncertainty of inactivation times for SARS-CoV-2 is large and depends on many factors including uncertainties of the experiments used to determine inactivation times and the matrix in which the virus is embedded. Based on the studies discussed above we conclude that 90% of SARS-CoV-2 virus particles will be inactivated by solar UV radiation within 4–20 min under optimal conditions for SZA ≤ 40°. These times will become considerably larger for SZA > 40°, during cloudy conditions, if surfaces are not directly irradiated by sunlight, or if virus particles are shielded from solar exposure by other means (absorption by matrix material, deposition on a porous material, shade). Even inactivation times as short as 5 min may be too long to protect against transmission among people in the outdoors talking to each other in close proximity. Furthermore, most transmissions occur indoors where there is essentially no exposure to solar UV-B radiation (most glass windows do not transmit at UV-B wavelengths), and both UV-A and visible solar radiation are greatly reduced, in particular when direct sunlight is blocked by window shades or other means. Hence, while solar radiation helps to disinfect surfaces or exhaled aerosol contaminated with SARS-CoV-2 particles, one cannot rely on the Sun’s germicidal effect in general and, in particular, early and late in the day, during winter, or at high latitudes during all seasons.

## Radiation amplification factors for SARS-CoV-2 action spectra

Radiation amplification factors (RAFs) are used to approximately assess the sensitivity of effects of UV radiation to changes in the stratospheric ozone layer. Specifically, the dimensionless RAF describes the relative change in effective UV irradiance (UV_BE_) in response to a relative change in TCO:

3 $${{\Delta {\text{UV}}_{{{\text{BE}}}} } \mathord{\left/ {\vphantom {{\Delta {\text{UV}}_{{{\text{BE}}}} } {{\text{UV}}_{{{\text{BE}}}} }}} \right. \kern-0pt} {{\text{UV}}_{{{\text{BE}}}} }} = - {\text{RAF}} \times {{\Delta {\text{TCO}}} \mathord{\left/ {\vphantom {{\Delta {\text{TCO}}} {{\text{TCO}}}}} \right. \kern-0pt} {{\text{TCO}}}}$$

where the symbol Δ expresses a change in UV_BE_ or TCO in absolute units. For example, a RAF of 1.5 means that a 1% change in TCO would lead to a 1.5% change in UV_BE_. For larger (> 10%) changes in TCO, such as the decreases in TCO that would have occurred without the implementation of Montreal Protocol, the power form of Eq. (3) [49] is typically applied, but the definition of the RAF remains unchanged:

4 $${{{\text{UV}}_{{{\text{BE}} + }} } \mathord{\left/ {\vphantom {{{\text{UV}}_{{{\text{BE}} + }} } {{\text{UV}}_{{{\text{BE}} - }} }}} \right. \kern-0pt} {{\text{UV}}_{{{\text{BE}} - }} }} = \left( {{{{\text{TCO}}_{ - } } \mathord{\left/ {\vphantom {{{\text{TCO}}_{ - } } {{\text{TCO}}_{ + } }}} \right. \kern-0pt} {{\text{TCO}}_{ + } }}} \right)^{{{\text{RAF}}}}$$

where the subscripts (+ and –) refer to the cases with higher or lower values of ozone and UV_BE_, respectively.

**Fig. 2:**
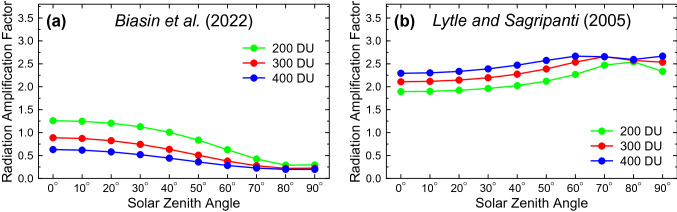
Radiation amplification factors for the action spectra by (**a**) Biasin et al. [23] and **b** Lytle and Sagripanti [24]. RAFs are shown as a function of SZA for TCO of 200, 300, and 400 Dobson Units (DU)

RAFs for the action spectra by Biasin et al. [23] and Lytle and Sagripanti [24] are shown in Fig. 2. RAFs for the action spectrum of Biasin et al. [23] range between 0.3 and 1.3 for SZA ≤ 60° and TCO between 200 and 400 DU, which cover the majority of conditions outside the polar regions. The magnitude and pattern is similar to RAFs for the erythemal action spectrum [28], which is expected given the similarity of the two action spectra (Fig. 1). In contrast, RAFs for the action spectrum of Lytle and Sagripanti [24] are between 1.9 and 2.7 for the same range of SZAs and TCO, and similar to those of the DNA damage action spectrum [34]. Hence, the effect of changes in ozone is more than a factor of two larger on average for the spectrum by Lytle and Sagripanti [24] than for that by Biasin et al. [23].

## Observed relationships between UV radiation and COVID-19 incidence

Many observational epidemiological studies have demonstrated an inverse relationship between some metric of UV radiation and COVID-19 incidence rates [50–60]. Most of these studies have flaws as discussed in the following. Many papers also report an inverse correlation with temperature and humidity; however, these relationships are not further discussed in depth.

Gorman and Weller [54] review the potential of UV radiation to alter morbidity and mortality from COVID-19 based on more than 30 studies from many countries. The article is based on knowledge as of October 2020 (less than one year after the start of the pandemic) and concludes that inverse associations have been observed between measures of ambient UV radiation and COVID-19 incidence and mortality in most, but not all studies. Depending on the study, UV intensities were quantified by the UV Index^5^ (UVI); UV-A, UV-B, and UV-A+UV-B irradiance; and vitamin D-weighted UV irradiance. Gorman and Weller [54] also point out that many studies did not adjust for important confounders such as population demographics, temperature, and humidity.

Carleton et al. [53], Choi et al. [52], and Ma et al. [50] applied sophisticated statistical analyses and adjusted for several confounders to quantify the relationship between UV radiation and various measures of COVID-19 or SARS-CoV-2 activity. However, the three studies are based on UV fields from the ERA5 reanalysis [61], which are defined as the integration over wavelengths between 200 and 440 nm (https://docs.meteoblue.com/en/meteo/data-sources/era5). The contribution from the wavelength range 400–440 nm, which is in the visible part of the spectrum, accounts for almost 50% of radiative energy of the “UV” datasets used in these studies. Their conclusions that higher UV radiation dose is associated with lower COVID-19 growth rates are, therefore, questionable. Furthermore, the three studies analysed data for relatively short periods close to the start of the pandemic (Carleton et al. [53] used 1 January to 10 April 2020, Choi et al. [52] used 1 March 2020 to 13 March 2021, and Ma et al. [50] used 15 March to 31 December 2020). Public health policies (e.g. lockdowns, closing of borders, social distancing, and wearing of masks); strategies in managing the pandemic; and available treatments for COVID-19 changed rapidly during 2020. These changes are important confounding factors that are difficult to quantify. Statistical analyses based on data from many countries, as used by Carleton et al. [53] and Choi et al. [52], are further hampered by the heterogeneity of the policies enacted around the world. Nevertheless, the three studies report strong inverse associations between solar radiation in the 290–440 nm range and various COVID-19 metrics. For example, Carleton et al. [53] conclude that the *increase* in seasonal “UV” exposure between January and June 2020 lowered extratropical Northern Hemisphere COVID-19 growth rates by 7.4% ± 2.9% (± 1 standard deviation). Over the same period, the seasonal decline in exposure to UV radiation in the extratropical Southern Hemisphere raised growth rates by 7.3% ± 2.9%. However, they also conclude that UV radiation has a substantially smaller effect on the spread of the disease than social distancing policies. Ma et al. [50] found that the fraction of the reproduction number *R*_t_ (defined as the mean number of new infections caused by a single infected person), which are attributable to temperature, specific humidity, and UV radiation, were 3.73%, 9.35% and 4.44%, respectively. Hence, these meteorological factors account for a total 17.5% of *R*_*t*_.

Moozhipurath et al. [56] reported that an increase in the daily maximum UVI by one unit was associated with a 1.2% decline in daily growth rates of cumulative COVID-19 deaths. UVI data were downloaded from https://darksky.net/ without identifying their source. The analysis is based on data from 183 countries and the period 22 January 2020 to 8 May 2020. The caveats of using heterogeneous data from a short period as noted above also apply here. In a follow-on study, Moozhipurath and Kraft [55] posit that reduced exposure to solar UV radiation during lockdowns, with people confined to their homes, reduced their vitamin D status (Sect. 6). This implies that the positive effect of lockdowns in reducing transmissions is partly offset by a greater risk of severe illness once an infection occurs. The authors conclude that lockdowns in conjunction with adequate exposure to UV-B radiation might have reduced the number of COVID-19 deaths more strongly than lockdowns alone, and estimate that there would be 11% fewer deaths on average with sufficient exposure to UV-B irradiation during the period when people were recommended not to leave their house. However, a major weakness of the study is the lack of measurement of the vitamin D status at the population level. The vitamin D status was instead estimated from UVI data used in their earlier study [56] without explicitly describing the assumed relationship between UVI and vitamin D status and without taking behavioural changes into account. For example, there is no evidence that lockdowns reduced vitamin D status at the population level. Indeed the opposite may have occurred in some countries due to reduced office hours. Of note, according to the paper’s “competing interests” statement, the lead author of the paper is a “full-time employee of a multinational chemical company involved in vitamin D business and holds shares in the company,” which may have biased the study.

Isaia et al. [60] correlated death rates and incidence rates of infections against vitamin D-weighted UV irradiation, fraction of people in nursing homes, air temperature, and comorbidities across Italy. The study is based on the short period of 25 February to 31 May 2020 when policies and treatment options rapidly evolved, coinciding with seasonal increase in UV radiation. The authors found that the amount of solar UV radiation contributed the most to the observed correlation, explaining up to 83.2% of the variance in COVID-19-affected cases per population. This very high percentage contradicts the much lower numbers given in the studies discussed above and is not reconcilable with the fact that most COVID-19 transmissions occur indoors. While Isaia et al. [60] speculate that the effect of UV radiation is mediated by the synthesis of vitamin D, vitamin D status of the population was not assessed. Considering that vitamin D status is also influenced by individual behaviour (sun exposure, sun protection, and supplementation), the study merely presents a hypothesis.

The inverse correlation between UV radiation and COVID-19 incidence or deaths documented in the studies above is qualitatively consistent with the virucidal effect of UV radiation and its role in raising 25(OH)D levels. However, each of these studies has major limitations and none of them provides a clear causative pathway to explain the observed associations quantitatively.

**Fig. 3:**
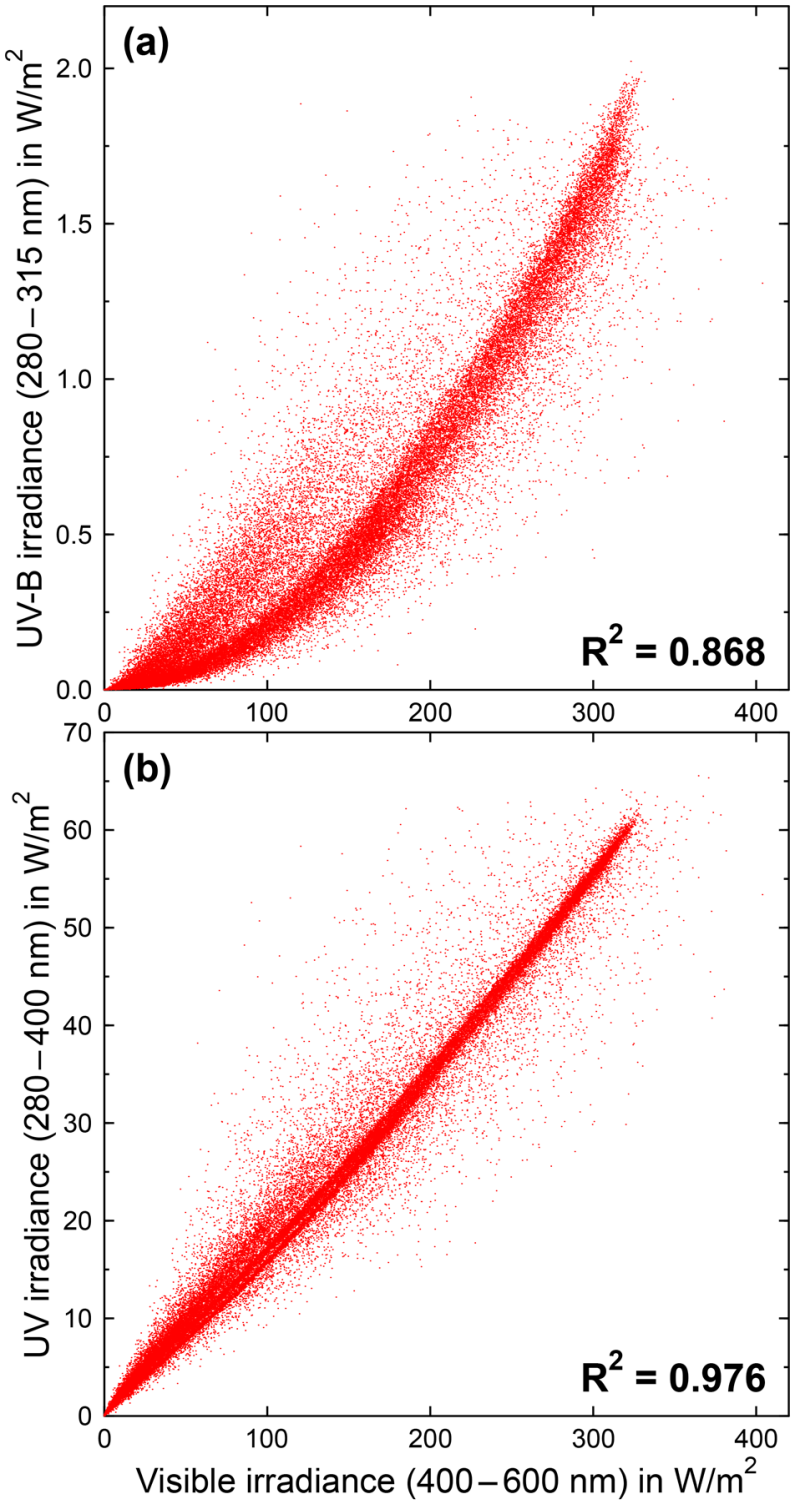
Scatter plot of (**a**) UV-B irradiance (280–315 nm) and **b** UV irradiance (280–400 nm) versus visible irradiance (400–600 nm). Data were measured in San Diego (32° N) between 2005 and 2008 with a SUV-100 spectroradiometer of the former National Science Foundation’s (NSF) UV monitoring network [68]. The coefficient of determination *R*^2^ is 0.868 for (**a**) and 0.976 for (**b**). Some of the scatter is caused by the fact that the SUV-100 is a scanning instrument, which requires about 15 min of time to complete a spectrum between 280 and 600 nm. Cloud conditions may change over this period, thereby exacerbating the scatter between the two quantities. If the whole spectrum had been measured at the same time, the scatter between the quantities would be smaller and *R*^2^ larger, suggesting that the actual covariance between UV and visible irradiance is even higher than indicated in the two plots

UV radiation strongly covaries with visible radiation, and it is, therefore, very difficult to determine from observational studies like those cited above whether the perceived seasonality is driven by UV radiation (e.g. via its germicidal effects or the production of vitamin D); visible radiation, which controls the circadian and circannual rhythms; or other factors that co-vary with UV radiation, such as temperature. This assertion is demonstrated in Fig. 3, which correlates measurements of UV-B and UV irradiance at San Diego, California (32° N), with visible (VIS) irradiance in the 400–600 nm range. The coefficients of determination *R*^2^ are 0.868 for UV-B vs VIS and 0.976 for UV vs VIS, suggesting that 86.8% and 97.6% of the variance in UV-B and UV irradiance, respectively, can be explained by the variance in visible irradiance. Compared to these strong associations, in particular for UV vs VIS, UV radiation and COVID-19 incidence rates are far less associated with each other; hence, studies that show a strong inverse correlation between the two variables would likely also show a strong inverse correlation between COVID-19 and visible irradiance even though visible radiation has little effect on virus survival and does not lead to vitamin D production.

Cherrie et al. [62] demonstrated a significant negative association between deaths from COVID-19 and ambient UV-A radiation and attribute this relationship to the release of nitric oxide (NO) from the skin upon exposure to UV-A radiation. Such release of NO has been shown to be associated with lower blood pressure [63] and reduced incidence of myocardial infarctions [64]. As there is evidence that hypertension and cardiometabolic disease also increase the risk of death from COVID-19 [65], Cherrie et al. [62] argue that UV-A-driven release of NO reduces the mortality from COVID-19. In a commentary to the paper, McKenzie and Liley [66] point out that the data could equally be explained by production of vitamin D by exposure to solar UV-B radiation considering the strong correlation between UV-A and UV-B radiation. Interestingly Guasp et al. [57] show a weaker inverse correlation between COVID-19 incidence and the UVI than with short-wave irradiance (300–3000 nm; a wavelength range including UV and visible radiation plus a part of the infrared spectrum). While this study is based on data from the very beginning of the pandemic only, it suggests that visible radiation may be a stronger determinant of disease incidence than UV radiation for reasons discussed in more detail below.

When interpreting the studies cited above, it has to be noted that correlation does not imply causation. For example, Martinez [67] pointed out that the incidence of polio, which peaks in the summer, correlates with temperature, daylength, and the sale of bathing suits. A transmission model using any of the three drivers would capture the seasonal structure of the disease because all drivers contain a covariate with the necessary seasonal dependence, even though it is highly unlikely that sales of bathing suits cause polio. Applying this thought experiment—combined with the high covariance between UV and visible radiation—to the observed inverse correlations between UV radiation and COVID-19 incidence rate suggests that these correlations cannot prove that UV radiation is the causative factor for the perceived seasonality of COVID-19.

The pilot phase of a prospective, double-blinded, randomised, placebo-controlled trial involving 30 patients has recently been completed that aimed to determine whether phototherapy with narrow-band UV radiation (NB-UVB) at 311 nm has an effect on the mortality of hospitalised, high-risk COVID-19 patients [69]. The trial was motivated by the observation that NB-UVB treatment stabilises the immune system relevant to autoimmune diseases. Considering that COVID-19 morbidity and mortality are partly driven by poor immune regulation (Sect. 6), it is, therefore, conceivable that treatments with NB-UVB may affect COVID-19 disease outcomes. The 30 enrolled patients were randomised 1:1 to NB-UVB or placebo phototherapy and treated daily with sub-erythemal doses for up to eight consecutive days. Although this pilot study was primarily to test safety and feasibility, it found that the 28-day mortality was 13.3% (2 patients) in the treatment and 33.3% (5 patients) for those receiving the placebo. However, the difference was not statistically significant (*p* = 0.39). Results for a planned follow-on study with a larger sample size are not yet available. If such a larger study were to demonstrate the efficacy of NB-UVB treatment, the effect of UV radiation on COVID-19 disease outcome could be firmly established. Such an outcome would also inform the assessment of the effects of solar UV radiation on COVID-19.

After many years of research, the factors that drive the seasonality of common diseases (and the relative importance of the factors causing seasonality) have still not been unambiguously determined. For example, humidity, temperature, closeness of people, changes in diets, and vitamin D status have been suggested to explain why influenza is more prevalent in winter than summer [15]. In addition, the human immune system may change with season, becoming more resistant or more susceptible to different infections based on daylength, and the disease burden is, therefore, partly driven by the circannual rhythm [70], which is, in turn, partly driven by visible radiation. For example, in one German cohort, expression in white blood cells of nearly one in four genes in the entire genome differed between seasons. Genes in the Northern Hemisphere tended to switch on when they were switched off south of the Equator, and vice versa [71]. Other factors that are potentially responsible for seasonality include: pathogen survival in the environment and transmissibility; changes over time in pathogen reservoirs (human and non-human); frequency of pathogen-host interactions (cultural, socioeconomic, linked to lifestyle and temperature); host susceptibility to infection; indoor heating systems that generate conditions of low relative humidity; and difference between indoor and outdoor temperatures [54, 70, 72–75]. Many of these factors co-vary with UV radiation. Diseases from enveloped viruses, such as SARS-CoV-2 and influenza viruses, generally have a stronger seasonality than those from viruses without an envelope, such as the rhinoviruses that cause the common cold [15]. It is, therefore, plausible that the factors that drive the seasonality of influenza are also predominantly responsible for the seasonality of SARS-CoV-2.

As mentioned in the introduction (Sect. 1), there is strong evidence that most SARS-CoV-2 infections occur indoors [13] where there is essentially no exposure to solar UV-B radiation. Furthermore, exposure studies have shown that adults working outdoors receive only about 10% of the total available annual UV radiation dose, while indoor-working adults and children get only about 2–4% of the available UV dose [76, 77]. These fractions were likely even lower during lockdowns. Despite the observed inverse correlations between UV radiation and COVID-19 incidence, and after considering the many other factors that correlate with UV radiation discussed above, we conclude that it is premature to establish UV radiation as the key factor driving the seasonality of the COVID-19 pandemic.

## Vitamin D and risk and severity of COVID-19

Vitamin D has important modulatory effects on the immune system that might reduce the risk and severity of COVID-19. The active form of vitamin D upregulates innate immunity by stimulating release of antimicrobial peptides, such as cathelicidin, which leads to an early defence against infection [78]. Vitamin D also influences the adaptive immune system, dampening down overproduction of pro-inflammatory cytokines, which can result in severe complications and organ damage (a cytokine storm). Vitamin D might also influence outcomes from COVID-19 through effects on the renin angiotensin aldosterone system, with important effects on vascular function, hypertension and cardiovascular remodelling [79]. Observational studies [80] and randomised controlled trials [81, 82] suggest a beneficial effect of vitamin D supplementation on the incidence and severity of acute respiratory tract infection, but there is relatively limited high-quality information about SARS-CoV-2 infection and/or COVID-19.

Meta-analyses of observational studies indicate inverse associations between 25(OH)D concentration and risk of SARS-CoV-2 positivity or COVID-19 disease or severity [83–86]. However, the quality of the observational studies has largely been low and heterogeneity high. Notable potential biases relate to lack of adequate control of confounders, self-reported SARS-CoV-2 positivity, and measurement of 25(OH)D concentration many years before the SARS-CoV-2 outbreak. In addition, a range of assays, with potentially variable accuracy and precision, have been used to measure 25(OH)D concentration; thus, even if the association is causal, an optimal 25(OH)D concentration cannot be defined.

Mendelian randomisation (MR) studies can overcome the bias introduced by confounding factors and timing of measurement. These studies assess associations between genetically determined, rather than measured, 25(OH)D concentration. The relatively small proportion of variability in 25(OH)D concentration explained by genetic variants necessitates a large sample size to give sufficient statistical power to detect small effect sizes. There have been two MR studies conducted within the COVID-19 host genetics initiative [87, 88]. The sample size and genetic instruments used varied somewhat, but both arrived at the same conclusions; that is, there is no statistically significant evidence of a causal effect of 25(OH)D concentration on COVID-19 susceptibility or severity, but small effects cannot be ruled out. In the larger of the two studies (total cases 17,964) [88], the odds ratio (OR) for risk of infection for each standard deviation increase in 25(OH)D (using the genetic instrument that explained the most variability) was 1.04 (95% CI 0.92, 1.18). The OR for severe disease (*n* = 4336 cases) compared with population controls was 0.96 (95% CI 0.64, 1.43). While there was no association with having 25(OH)D concentration < 50 or < 75 nmol/L (commonly used cut-points to define vitamin D deficiency or insufficiency, respectively) [88], a link with more severe vitamin D deficiency (e.g. 25(OH)D < 30 nmol/L) cannot be excluded.

Several randomised trials have examined the effect of supplementing hospitalised COVID-19 patients with vitamin D on disease outcomes, with heterogeneous findings. A randomised placebo-controlled trial in Brazil supplemented patients (*n* = 240) with a large single oral dose of 200,000 IU of vitamin D_3_ or placebo^6^ [89]. There was no difference in the length of stay (primary outcome; median 7 days in both groups) and no statistically significant difference in any of the secondary outcomes, specifically in-hospital mortality (vitamin D 7.6% vs placebo 5.1%; *p* = 0.43), admission to the intensive care unit (16.0% vs 21.2%; *p* = 0.30) or mechanical ventilation (7.6% vs 14.4%; *p* = 0.09). Similarly, a study in Argentina did not find any benefit of high-dose vitamin D supplementation [90]. In this multicentre randomised controlled trial, 218 hospitalised patients with confirmed COVID-19, mild-to-moderate symptoms, and risk factors for progression were randomised to a single oral dose of 500,000 IU of vitamin D_3_ or placebo. There was no significant effect on the respiratory score of Sepsis-related Organ Failure Assessment (*p* = 0.93), in-hospital mortality (4.3% vitamin D vs 1.9% placebo, *p* = 0.45) or other secondary outcomes (length of stay and intensive care unit admission). In contrast, a pilot trial in Spain, in which 76 hospitalised patients were randomised to control (usual care) or supplementation with 0.532 mg of 25(OH)D (calcifediol) on the day of admission,^7^ day 3, and day 7, followed by a weekly dose of 0.266 mg until discharge, observed reduced admission to intensive care in the supplemented group (2% vs 50%; *p* < 0.001) [91]. An open-label trial^8^ in France, in which 254 hospitalised patients aged ≥ 65 years were randomised to a single oral dose of 400,000 IU or 50,000 IU of vitamin D_3_, found reduced deaths at 14 days in the high-dose group (6%) compared with the lower-dose group (11%) (*p* = 0.049) [92]. In addition to the inconsistency in the findings, and some limitations related to trial design in some studies, all used very large bolus doses (i.e. a single dose given all at once) of vitamin D, which are largely uninformative about the effects of vitamin D obtained through sunlight or usual supplementation doses.

In conclusion, the evidence supporting a role of vitamin D in the risk or severity of COVID-19 is currently inconsistent. In addition, there is limited information about the effect of severe vitamin D deficiency. Given the laboratory evidence supporting a role of vitamin D in the immune system, and the indications of benefit for other acute respiratory tract infections, it would be prudent to adopt a precautionary principle and develop policies to avoid vitamin D deficiency. However, routine supplementation of populations that are not experiencing vitamin D deficiency is currently not warranted.

## Effect of ambient air pollution and SARS-CoV-2 infections

Ambient air quality is affected by pollutants that are emitted into the air. Photochemical smog is formed when these pollutants are exposed to solar UV radiation [93]. Several studies reported a decrease in air pollution following the enforcement of lockdowns in many countries worldwide resulting in a reduction of excess mortality [94, 95]. The progression of the COVID-19 pandemic may also have been affected by changes in ambient air pollution. A study that analysed the effect of air pollution on the 2002–2004 outbreak of severe acute respiratory syndrome (SARS), a disease which predated COVID-19 and was caused by the severe acute respiratory syndrome coronavirus 1 (SARS-CoV-1), yielded mixed and inconclusive results [96]. On the other hand, several recent studies indicate significant associations between air pollution and COVID-19 incidence and fatality rates [97–100]. While factors associated with infection have been well established, such as the proximity to infected persons in indoor spaces through airborne transmission [101], the question of the role of ambient air pollution in the spread of SARS-CoV-2 and the development of the pandemic is still unclear. For the USA, Wu et al. [102] found that higher historical exposures to PM_2.5_ (particles with a diameter of ≤ 2.5 µm) were positively associated with higher county-level COVID-19 mortality rates after accounting for many area-level confounders. Specifically, an increase of 1 µg m^−3^ in the long-term average of PM_2.5_ was associated with a statistically significant 11% (95% CI 6%, 17%) increase in the county’s COVID-19 mortality rate. Similarly, by analysing data from China for the period 19 January 2020 to 15 March 2020 and applying a lag of 21 days between COVID-19 diagnosis and death, Yao et al. [103] found that a higher case-fatality rate of COVID-19 was associated with higher (historical, 2000–2016) daily concentrations of PM_2.5_ and PM_10_ (particles with a diameter of ≤ 10 µm). There are several biological pathways that have been proposed whereby exposure to outdoor air pollution may relate to transmission, host susceptibility, and disease severity [104, 105]. Air pollution has been postulated to affect the viability and transport of viral particles in the air [106] and hence increase respiratory infections. It is also possible that air pollution could increase severity of COVID-19 through its contribution to chronic conditions—such as chronic respiratory disease, diabetes, and heart disease—and through long-term effects on immune system function [107]. However, the first results in well-established cohort studies indicate no direct association between exposure to air pollution and infection with SARS-CoV-2 [108]. Hence, the role of air pollution in the transmission and severity of the disease is still not well established and results from epidemiological and experimental studies that could elucidate this issue further are still lacking.

## Link between the Montreal Protocol and the inactivation of SARS-CoV-2

While the Montreal Protocol has prevented run-away increases in solar UV radiation [Sect.  4.1 of 25], it may have also affected the inactivation rate of pathogens exposed to UV radiation. According to McKenzie et al. [109], the Montreal Protocol has averted increases of erythemal (sunburning) irradiances by approximately 20% between the early 1990s and 2018 at mid-latitudes. Under the presumption that the action spectrum measured by Biasin et al. [23] is correct, the sensitivity to changes in TCO should be similar for erythemal irradiance and the effective irradiance for the inactivation of SARS-CoV-2 virus particles (Sect. 4). This conclusion implies that inactivation times of SARS-CoV-2 viruses would be about 20% shorter today if the Montreal Protocol had not been implemented. However, a larger effect would be expected if the actual action spectrum were closer to that reported by Lytle and Sagripanti [24]. Whatever the actual sensitivity to changes in TCO may be, it is unlikely that this effect has any tangible consequences on the progress of the COVID-19 pandemic considering that: (i) fomites in the outdoors that are exposed to solar UV radiation provide the least likely mode of transmission; (ii) outdoor infections via exhaled droplets or aerosol are the exception; (iii) outdoor transmission in the few cases that have occurred were likely between people talking or acting in close proximity where inactivation times even in full sunlight are too long to have a significant effect; and (iv) the role of UV-B radiation in raising 25(OH)D levels, which may protect from severe disease progression, have not been convincingly documented (Sect. 6). On the other hand, the far-reaching, positive outcomes of the successful implementation of the Montreal Protocol for life on Earth [25, 93, 110–114] outweigh any potential advantage for disinfection by higher amounts of solar UV radiation.

## Gaps in knowledge

Our assessment identified the following gaps in knowledge:

- To date, the action spectrum for the inactivation of SARS-CoV-2 has been measured by only one group [23] and the experiment for establishing this spectrum has several weaknesses (Sect. 2). Furthermore, this action spectrum has a relatively large contribution from wavelengths in the UV-A range, in contrast to action spectra published for many other viruses [24]. The reason for this discrepancy is presently unknown and the measurements by Biasin et al. [23] have not been independently confirmed.
- Several groups have measured inactivation times of SARS-CoV-2 upon exposure to simulated UV radiation. While all studies confirmed the germicidal effects of UV radiation, the measured inactivation times vary widely and depend on many factors, including experimental setup, sample preparation, and the medium in which the sample is embedded (e.g. saliva, growth medium, or aerosol). Inactivation times have not been measured yet under solar radiation and the extrapolation of the laboratory studies to real-world settings is, therefore, subject to large uncertainties.
- While many studies demonstrate inverse correlations between UV radiation and COVID-19 incidence or severity of disease, a convincing theory explaining these associations is still missing and the causative factors at play, and their relative contributions, are still unknown.
- It is not clear how research on the seasonality of common virus-caused diseases such as the common cold and influenza can be best applied to augment our understanding of the observed seasonality of COVID-19.
- While observational studies indicate inverse associations between 25(OH)D concentration and risk of SARS-CoV-2 positivity or COVID-19 severity, Mendelian randomisation studies have not found statistically significant evidence of a causal effect of 25(OH)D concentration on COVID-19 susceptibility or severity. Furthermore, randomised trials have generated mixed results. Hence, there is currently no reliable quantification of the role of vitamin D in reducing the susceptibility or severity of COVID-19.
- While several observational studies indicate significant associations between air pollution and COVID-19 incidence and fatality rates, results of well-established cohort studies indicate no association between long-term exposure to air pollution and infection with SARS-CoV-2. Hence, the role of air pollution in the transmission and severity of disease is still not well established.

## Conclusions

By preventing large increases in UV radiation, the Montreal Protocol may have also affected the inactivation rates of SARS-CoV-2 exposed to solar UV radiation. Without the Montreal Protocol, these rates would have been larger; however, it is unlikely that this would have significantly changed the progression of the COVID-19 pandemic. The most reliable experimental data suggest that 90% of SARS-CoV-2 particles are inactivated by solar radiation within ~ 7 min for high sun and ~ 13 min for low sun. However, one cannot rely on the Sun’s germicidal effect in general and, in particular, early and late in the day, during winter, or at high latitudes during all seasons. There is evidence of an inverse relationship between ambient solar UV radiation and the incidence or severity of COVID-19, but the reasons for this inverse correlation have not been unambiguously identified as they can also be explained by confounders, such as ambient temperature, humidity, visible radiation, daylength, temporal changes in risk and disease management, and the proximity of people to other people. Observational studies indicate that higher concentrations of vitamin D (specifically 25(OH)D) in the blood are correlated with lower risk of SARS-CoV-2 positivity or severity of COVID-19. While reasons for this inverse relationship have not been established, a potential link between vitamin D status and disease severity cannot be excluded at this time. Considering that laboratory studies support the role of vitamin D in the immune system, it would be prudent to advocate policies to avoid vitamin D deficiency. However, routine supplementation of populations that are not experiencing vitamin D deficiency is currently not warranted.

Assessments on the effect of solar UV radiation on COVID-19 prevalence and severity are impeded by: the uncertainty of the action spectrum for the inactivation of SARS-CoV-2; the lack of measurements of inactivation rates of SARS-CoV-2 under solar radiation; the dearth of controlled clinical trials investigating the causes of the inverse association between ambient UV radiation and incidence or severity of COVID-19, which is indicated by observational studies; and the inconsistency between the results of observational studies and randomised trials concerning the role of vitamin D and air pollution in the incidence and progression of COVID-19.

## Data Availability

All data generated or analysed are either included in this published article or are part of the analyses of papers cited.

## Footnotes

1 A negative correlation expresses a statistical relationship between two variables whereby higher values of one variable tend to be associated with lower values of the other. Several essentially equal terms for “negative correlation” are being used, including anticorrelation, inverse correlation, and negative (or inverse) association.

2 Ozone amounts integrated from the Earth’s surface to the top of the atmosphere, measured in Dobson Units (DU). See [25] for more details.

3 Glasgow’s Minimum Essential Medium.

4 Illuminance is expressed in the unit of lux (lx) and is the spectral irradiance weighted with the sensitivity of the human eye under daylight conditions, expressed by the photopic luminosity function, and scaled with 683.002 lx W^−1^ m^2^.

5 The UV Index is calculated by weighting solar UV spectra with the action spectrum of erythema [28] and scaling the result with 40 m^2^/W.

6 For comparison, the recommended daily intake of vitamin D_3_ ranges from 400 to 800 IU/day in many countries.

7 For comparison, the typical recommended dose in clinical settings is 50–100 µg (0.05–0.1 mg).

8 An open-label trial is a medical study in which both investigators and trial participants are fully aware of which treatment group the participants are in and what treatments are assigned to them.
